# Melanoma arising from partially biopsied moderately dysplastic nevus

**DOI:** 10.1016/j.jdcr.2025.01.040

**Published:** 2025-03-07

**Authors:** Jonathan C. Hwang, Bryan L. Peacker, Christine G. Lian, Eleanor E. Russell-Goldman, Christine M. Cornejo, Frank R. Vleugels, Rebecca I. Hartman

**Affiliations:** a University of Pittsburgh School of Medicine, Pittsburgh, Pennsylvania; b Department of Dermatology, Brigham and Women’s Hospital, Boston, Massachusetts; c Harvard Medical School, Boston, Massachusetts; d Department of Pathology, Brigham and Women’s Hospital, Boston, Massachusetts; e Dermatology Section, VA Boston Healthcare System, Jamaica Plain, Massachusetts

**Keywords:** dysplastic nevus, melanoma, melanoma transformation, moderately dysplastic nevus, partial biopsy, positive margins, skin cancer

## Introduction

Dysplastic nevi (DN) are atypical melanocytic lesions histopathologically situated between benign melanocytic nevi and cutaneous melanoma.^1^ Although DN are a risk factor for melanoma, only a few nevi progress to melanoma at the same site. Transformation rates range from 1 in 9735 melanocytic nevi for males and 1 in 12,880 melanocytic nevi for females.^2^ There is a stronger consensus regarding the management of both mild and severe DN with positive margins; mild DN generally do not require re-excision, whereas severe DN typically warrant excision due to higher risk of malignant transformation and misdiagnosis.^1^^,^^3^ However, the management of moderate DN has been a subject of considerable debate and variability in clinical practice.^3^ The Pigmented Lesion Subcommittee of the Melanoma Prevention Working Group released a consensus statement that observation of moderate DN with positive histologic margins and no clinical pigmentation is reasonable, but did not provide definitive recommendations due to insufficient data.^1^ A 2018 study supported this recommendation, reporting no cases of melanoma at the biopsy site for 467 moderate DN with positive histologic margins that had no grossly observed residual pigment.^4^ We report a case of a patient with a moderate DN with positive margins and no residual pigmentation who subsequently developed a pink papule at the biopsy site consistent with melanoma.

## Case report

**Fig 1:**
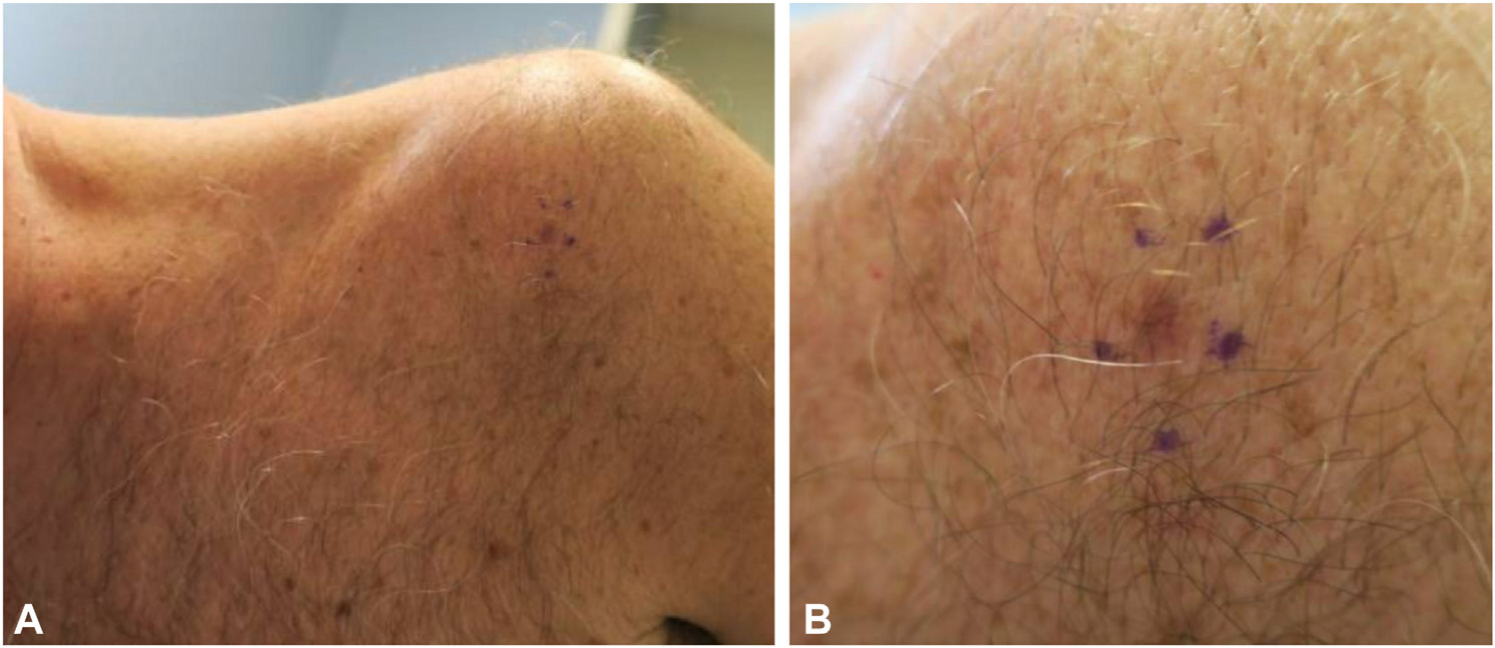
**A,***Light brown* papule with peripheral erythema on the patient’s left shoulder prior to initial shave biopsy. **B,** A close-up view of the papule.

A 49-year-old man presented for a routine skin examination. His medical history included a superficial basal cell carcinoma on the mid-back managed with electrodesiccation and curettage 5 months prior. He reported extensive ultraviolet exposure in youth. He had no family history of melanoma. On examination, an asymptomatic pink and brown thin macule was noted on his left shoulder (Fig 1).

Clinical examination favored basal cell carcinoma. A shave biopsy revealed a lentiginous compound DN with moderate atypia of the intraepidermal component and positive margins, extending to the tissue edge. No pagetoid spread was observed, and the nevic component contained small nevoid cells. Upon repeat skin examination 3 months later, no concerning lesions or repigmentation of the biopsy site were noted.

**Fig 2:**
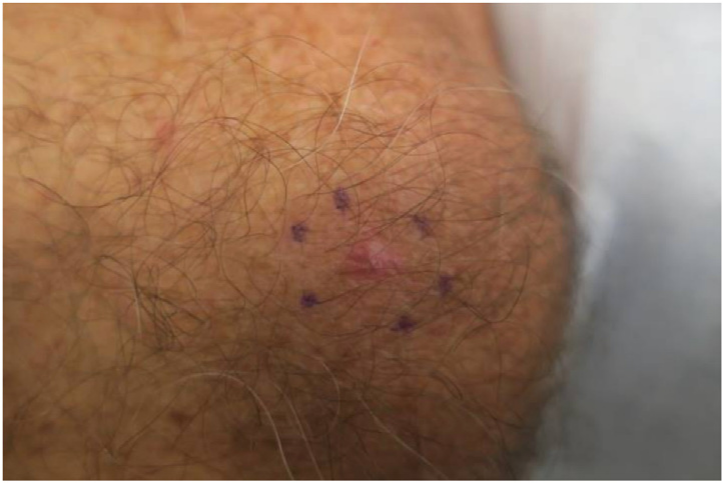
*Shiny pink* papule adjacent to the initial biopsy scar, 6 months postbiopsy. A subsequent shave biopsy of this pink papule confirmed the diagnosis of malignant melanoma.

Six months after the initial biopsy, a shiny pink papule was noted adjacent to the original biopsy scar (Fig 2).

**Fig 3:**
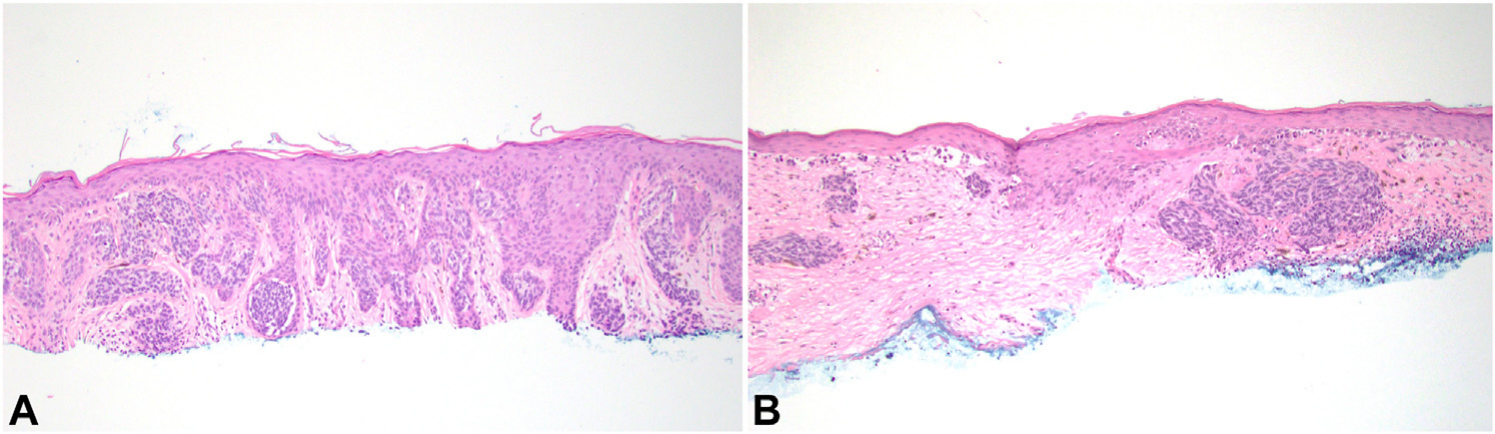
**A,** Initial biopsy showed a compound dysplastic nevus with a nested junctional component with bridging and concentric lamellar fibroplasia around rete ridges. The dermal component was composed of small bland epithelioid melanocytes and showed maturation with depth (hematoxylin and eosin stain, 100× magnification). **B,** Subsequent biopsy revealed melanoma. In contrast to the prior sample, this biopsy showed melanoma in situ with predominantly lentiginous and contiguous growth and pagetoid spread of the junctional component. The invasive melanoma component was composed of large atypical epithelioid to spindled cells, distinct from those seen in the nevus component (hematoxylin and eosin stain, 100× magnification).

Repeat shave biopsy revealed a confluent atypical junctional melanocytic proliferation with pagetoid spread, consistent with malignant melanoma with Breslow thickness 0.4 mm, Clark level III, with 0 mitoses per mm^2^ (Fig 3).

The invasive component exhibited large epithelioid and spindled melanocytes. No lymphovascular invasion or neurotropism was identified. The lesion was present at the side margin and focally involved the deep margin. Each specimen was reviewed by a board-certified dermatopathologist to confirm the diagnosis. The melanoma was also presented at the Brigham and Women’s Hospital melanoma consensus conference for further validation. The patient was diagnosed with malignant melanoma arising adjacent to the prior site of a moderate DN and underwent wide local excision. There has been no recurrence to date over the past 4 years.

## Discussion

The management of moderately atypical DN with positive histologic margins without residual pigmentation remains a controversial area in dermatologic practice. Although Pigmented Lesion Subcommittee recommendations tentatively support observation in such cases, substantial variability exists in clinical practice.^2^ 52% of pigmented lesion clinic directors recommend no additional procedure, while 48% recommend additional margins of between 1 and 4 mm after initial biopsy.^3^ This variability is further compounded by low intraobserver reproducibility, with histopathologic agreement at 41% for moderate DN and 47% for severe DN.^4^

Only a few cases have reported transformation of nevi to melanoma (Table I).

**Table I:** Reported cases of melanoma arising from benign, mild, and moderately atypical nevi

Case	Age, sex	Initial clinical information	Initial biopsy findings	Positive margins?	Time to melanoma	Melanoma clinical information	Melanoma biopsy findings	Outcome
Helm et al, 2021^5^	33, F	Periumbilical pigmented lesion	Benign nevus, no residual lesion	No	16 mo (during pregnancy)	Asymmetric pigmented lesion extending beyond scar, “persistent nevus”	Clark level IV, Breslow depth 0.8 mm, 1 mitosis/mm^2^	Wide excision; no recurrence to date of report (4 y)
Jeong et al, 2019^6^	35, F	Left cheek	Recurrent (x3) moderate DN	No	5.75 y	Left cheek, left temporal, right retroauricular, and left shoulder	Stage IV (T4aN0M1a) SSM, Breslow depth 6 mm (2-8 mm other sites)	2 mo later diagnosed with MIS on forehead; chemotherapy with high-dose interferon with no recurrence to date (3 y)
Fleming et al, 2016^7^ (case 788)	NR	6-mm light tan macule with irregular pigmentation on 1 border	JMN with mild-moderate dysplasia, transected laterally	Yes	5 y	Tan papule with speckled pigmentation (12 mm)	Invasive melanoma (LM type), Breslow depth 0.8 mm	Wide excision; no patients died of melanoma arising from DN biopsy sites
Fleming et al, 2016^7^ (case 917)	NR	1.5-cm flat brown patch with minimal color variegation	JMN with mild-moderate dysplasia, transected laterally	Yes	10 y	Brown/black patch with several foci of darker pigment (2.2 × 2-cm)	Early MIS (LM type) arising in a JMN with moderate-to-severe dysplasia	Wide excision; no patients died of melanoma arising from DN biopsy sites
Fleming et al, 2016^7^ (case 1158)	NR	12 × 10-mm darkly pigmented patch	JMN with mild-to-moderate dysplasia, transected laterally	Yes	2 y	Dark pigmented nevus (10 × 12 mm)	MIS (LM type)	Wide excision; no patients died of melanoma arising from DN biopsy sites
Fleming et al, 2016^7^ (case 1197)	NR	10 × 9-mm pigmented patch with a focus of darker pigmentation	JMN with mild-to-moderate dysplasia, transected laterally	Yes	3 y	Darker pigmentation within residual lesion	MIS (LM type)	Wide excision; no patients died of melanoma arising from DN biopsy sites
Fleming et al, 2016^7^ (case 1871)	NR	11 × 7-mm atypical pigmented patch	JMN with mild-to-moderate dysplasia, narrowly excised	Yes	1 y	Residual pigmentation around biopsy scar	MIS (LM type)	Wide excision; no patients died of melanoma arising from DN biopsy sites
Fleming et al, 2016^7^ (case 456)	NR	1-cm mostly round dark brown patch	JMN with moderate-focal severe dysplasia, no residual lesion	Yes	9 y	Hyperpigmented patch with darker periphery and central lighter brown color	MIS (ALM type)	Wide excision; no patients died of melanoma arising from DN biopsy sites
Current case	49, M	0.8 × 0.4 cm light brown papule with peripheral erythema, left shoulder	Lentiginous compound moderate DN, positive margins	Yes	6 mo	Shiny pink papule adjacent to original biopsy scar on left shoulder	Malignant melanoma, Breslow depth 0.4 mm, Clark level III, 0 mitoses/mm^2^	Wide excision; no recurrence to date (4 y)

*ALM*, Acral lentiginous melanoma; *DN*, dysplastic nevi; *F*, female; *JMN*, junctional melanocytic nevus; *LM*, lentigo maligna; *M*, male; *MIS*, melanoma in-situ; *NR*, not reported; *SSM*, superficial spreading melanoma.

A pregnant patient developed melanoma 16 months after a biopsy revealed a benign nevus with no residual lesion.^6^ Another case described recurrent moderate DN at the same site despite 3 complete re-excisions, which developed into malignant melanoma 5.75 years after the last re-excision.^6^ Although Kim et al (2018) found no malignant transformation among moderate DN in a large retrospective cohort study, the study size may be underpowered to detect rare events.^8^ An earlier retrospective cohort study with a larger sample size that included partial biopsies found 5 cases of melanoma arising from mild-to-moderate DN and 1 case arising from moderate DN among patients who were observed after initial biopsy.^7^ Five of the 6 melanoma cases arose from DN in which the initial biopsy was partial, leading the authors to recommend saucerization or deep shave biopsy to avoid missing melanoma. This present case is among the few reports of moderate DN with positive margins that showed no clinical repigmentation but later progressed to melanoma.^7^

One limitation of this case is that histologic assessment of moderate atypia inherently involves a degree of subjectivity. Even among board-certified dermatopathologists, intraobserver reproducibility for moderate DN can be as low as 41%.^4^ While misdiagnosis of the initial lesion as moderate DN rather than melanoma cannot be entirely ruled out, the original diagnosis was made by a fellowship-trained, board-certified dermatopathologist with expertise in melanocytic lesions. Another limitation is that MART-1/Melan-A staining was performed only on the specimen diagnosed as melanoma, and not on the initial moderate DN specimen for comparison. However, while MART-1/Melan-A staining is useful for identifying melanocytic cells, it lacks specificity both in distinguishing melanocytic from nonmelanocytic lesions and in differentiating between benign from malignant melanocytic lesions.^9^ Future studies should explore whether specific immunohistochemistry patterns correlate with progression risk, potentially improving management strategies for cases with diagnostic uncertainty.

This case highlights the low but potential risk of melanoma progression from moderate DN to melanoma. Larger studies could further clarify the rare possibility of malignant transformation, variability in histologic grading of atypia, and possibility of amelanotic recurrences.^10^ While postbiopsy surveillance often focuses on repigmentation at the biopsy site, it is important to recognize that recurrences can also present as amelanotic lesions.^1^ Clinicians and patients should be aware of continuing to monitor biopsy sites of moderate DN with positive histologic margins, even those without clinical repigmentation. Development of repigmentation or new pink lesions warrants further investigation regardless of the original lesion’s pigmentation.

## Conflicts of interest

None disclosed.
